# Variation in the Content and Composition of Tocols in a Wheat Population

**DOI:** 10.3390/foods11091343

**Published:** 2022-05-05

**Authors:** Karolina Tremmel-Bede, Marietta Szentmiklóssy, Anna-Maija Lampi, Vieno Piironen, Peter R. Shewry, Gyula Vida, Sándor Tömösközi, Ildikó Karsai, László Láng, Zoltán Bedő, Marianna Rakszegi

**Affiliations:** 1 Agricultural Institute, Centre for Agricultural Research, Brunszvik u. 2., 2462 Martonvásár, Hungary; bede.karolina@atk.hu (K.T.-B.); vida.gyula@atk.hu (G.V.); karsai.ildiko@atk.hu (I.K.); langlmv@gmail.com (L.L.); bedo.zoltan@atk.hu (Z.B.); 2 Research Group of Cereal Science and Food Quality, Department of Applied Biotechnology and Food Science, Budapest University of Technology and Economics, Műegyetem rkp.3, 1111 Budapest, Hungary; szentmiklossy.marietta@vbk.bme.hu (M.S.); tomoskozi.sandor@vbk.bme.hu (S.T.); 3 Department of Food and Nutrition, University of Helsinki, Agnes Sjöbergin katu 2, 00014 Helsinki, Finland; anna-maija.lampi@helsinki.fi (A.-M.L.); vieno.piironen@helsinki.fi (V.P.); 4 Rothamsted Research, Harpenden, Hertfordshire AL5 2JQ, UK; peter.shewry@rothamsted.ac.uk

**Keywords:** breeding, diversity, G × E, heritability, recombinant inbred lines, tocol, wheat

## Abstract

Wheat is a well-known source of B vitamins but also contains significant amounts of vitamin E and related tocols, which have a number of positive health benefits. However, there are no reports on increasing the tocol content of wheat. A prerequisite for increasing the tocol content is the identification of variation in its amount within wheat and related cereals. We therefore determined the tocol content and composition in the grain of 230 recombinant inbred lines (RILs) of a diverse biparental wheat population (Mv Toborzó/Tommi), showing variation in the total content from 13.69 to 45.18 μg/g d.m. The total content also showed transgressive segregation in the population. The effect of the genotype on the variance components of tocols was studied, and the broad-sense heritability was calculated to be 0.71. The lines were also grouped based on their tocol content and analyzed for their chemical composition and breadmaking quality. The high heritability value and the wide variation found in the total amount indicate that increasing the content of tocols is a possible breeding strategy.

## 1. Introduction

Wheat and other cereals are major sources of a range of components required for human nutrition and health, including dietary fiber, vitamins, and mineral micronutrients. Tocols are a group of terpenoids comprising a chromanol ring with a C16 phytol side chain. They occur in forms in which the side chain is either saturated (tocopherols) or has three double bonds (tocotrienols) with both tocopherols and tocotrienols existing in four related α, β, γ, and δ forms, which differ from each other by the number and position of the methyl groups on the chromanol ring. Tocols are often referred to as vitamin E but differ in their activity, with α-tocopherol being the most active form [1]. Currently, only α-tocopherol is considered to have vitamin E activity [2]. Cereals are rich sources of tocols, with the highest levels being in barley and soft wheats [3]. Most of the tocopherols (over 90% of all tocols) are located in the germ (~25 mg vitamin E/100 g in wheat) [4,5], but significant concentrations of tocopherols are also present in wheat bran [6]. Cereals (especially oats, rye, and barley) are also rich sources of tocotrienols [3,7,8].

Genetic and environmental factors have impacts on the contents and composition of wheat phytochemicals. A study of the concentrations of bioactive components (tocols, sterols, alkylresorcinols, folates, phenolic acids, and fiber components) in 26 wheat cultivars grown in a combination of 6 sites and years showed that the total tocol content is highly heritable, making it a suitable target for plant breeding [7,9]. However, there were significant correlations between the contents of the bioactive components and the environmental factors (precipitation and temperature) during grain development, and even the amount of highly heritable components varied between grain samples grown in different years and in growing areas [7]. The amounts of tocols, sterols, folates, stanols, and free and conjugated phenolic acids showed similar positive correlations with the temperature and negative correlations with the precipitation, resulting in negative correlations between these groups of components when samples grown under different climatic conditions were compared [7]. Shewry et al. [8] also determined the effect of the genotype and environment on the content of phytochemicals in rye. They found that the genotype had no significant effect on the total tocol content but had significant effects on some individual tocol components. No correlations were found between the total tocol content and the weather conditions, while in wheat, it was positively correlated with the temperature [7]. This difference may suggest that the tocol content is more stable in rye but may also mean that the smaller dataset (only 5 rye compared with 26 wheat lines) gave less significant correlations. The tocol content of some wheat genotypes was found to be more sensitive to the environmental effects [10]. The total tocol also showed a strong negative correlation with the thousand-kernel weight (r = −0.713) due to the higher proportion of bran in smaller grains, which is more concentrated in tocols [8].

An important chemical property of tocols is their antioxidant activity. Kinetic studies have shown that α-tocopherol is the most effective antioxidant, with the order of the reaction rate being the α, β, γ, and δ forms [11]. Tocotrienols may have similar or greater potential as antioxidants when compared with tocopherols [12,13]. The most important lipid-soluble antioxidant in the human body is vitamin E, which together with other antioxidants such as vitamin C protects the body against the effects of oxidative stress. Thus, the tocol content of cereals may reduce the risks of cancer and cardiovascular diseases and lower the low-density lipoprotein cholesterol level by inhibiting the biosynthesis of cholesterol [14]. Vitamin E has been linked to maintaining the immune system, relieving the symptoms of inflammatory diseases, and reducing the risk of cataracts and many other degenerative diseases [1,15]. It has recently been found that a high intake of vitamin E with food has reduced the incidence of Alzheimer’s disease [16]. Interestingly, both in vitro and in vivo studies have shown that tocols can reduce the acute effects of radiation on radiation-sensitive tissues in the hematopoietic and gastrointestinal tract, ultimately increasing the overall survival of victims exposed to radiation [17,18,19].

It is also important to consider the effects of processing on the tocol content, as both tocopherols and tocotrienols are readily oxidized, especially in the presence of heat, light, or alkali. Hidalgo and Brandolini [20] showed that the total tocol content decreased during the production of wheat-based foods. The process of kneading resulted in degradation in the bread and biscuits (on average, 21.4 and 28.2%, respectively), while the effect of leavening and baking the bread was almost negligible. During pasta making, the long kneading process caused high losses (44.2%), while the extrusion process resulted in an average decrease of 3.7% with the use of a vacuum, and a reduction of 29.7% was observed without the use of a vacuum. However, drying did not cause any significant loss. The average total losses during the whole production process were 24.1% and 25.4% in breadcrumbs and crust, respectively, 25.3% in water biscuits, and 41% and 55.7% in pasta with and without vacuum extrusion, respectively. Fratianni et al. [21] reported that pasta making did not appear to influence the bioaccessibility of tocols from durum wheat pasta, but there was a resulting 30% loss in the amount of tocols. Piironen et al. [22] and Wennermark and Jagerstad [23] concluded that the quality of the ingredients and the duration of dough making are the main factors contributing to the loss of tocols during baking, while the time and temperature of baking have a smaller effect.

In order to facilitate the increase in grain tocols by breeding, we determined their content and composition in 230 recombinant inbred lines (RILs) of a diverse biparental wheat population (Mv Toborzó/Tommi). We also calculated the effect of the genotype on the variance components and their broad sense heritability. The lines were then grouped based on the tocol content and analyzed to determine the correlation to the grain composition and breadmaking quality.

## 2. Materials and Methods

### 2.1. Development of Mv Toborzó/Tommi Wheat Recombinant Inbred Population

The Mv Toborzó variety was crossed with the Tommi cultivar to create a winter wheat population of 230 RILs through 8 cycles of self-fertilization. To produce the population, parental varieties were selected that were bred under different ecological conditions. Mv Toborzó is an early maturing Hungarian variety, while the German Tommi is a very late variety in Hungary. The two varieties carry different alleles in the PPD-D1 (2D) day-length sensitivity gene and the Rht-B1 (4B), Rht-D1 (4D), and Rht8 (2D) dwarf genes [24], so the heading date, plant height, and yield components of the RIL lines are highly variable. During the Healthgrain EU FP6 project, it was also found that Tommi contained high amounts of phytochemicals. For example, the contents of the sterols, tocols, and folates were high compared with the other 150 varieties studied [25], while Mv Toborzó contained a high amount of fiber. The compositional and breadmaking quality traits of the population were determined in 2 years (2014 and 2019). Six field replicates from the Mv Toborzó and Tommi controls were examined and averaged.

### 2.2. Field Conditions

The 230 lines were grown at the Agricultural Institute of the Agricultural Research Center in Martonvásár (latitude: 47°3′ N, longitude: 18°8′ E, altitude: 115 m) for 2 years (2014 and 2019). The plots were 2.5 m long and contained 6 rows spaced 20 cm apart. The soil had a clayey texture of chernozem and a pH of 7.25. The soil contained 2.8% *w*/*w* humus, 210 mg/kg P_2_O_5_, and 210 mg/kg K_2_O. The previous crop was oilseed radish (2013–2014 and 2018–2019). The yearly average N input as NPK combined fertilizer was a 120-kg/ha active ingredient (Table S1). The plots were treated with herbicide (4 L/ha U-46 D-fluid SL with 500 g/L 2-methyl-4-chlorophenoxyacetic acid and 40 g/ha Granstar 50 SX with 50% tribenuron methyl), insecticide (0.2 L/ha Karate Zeon 5CS, containing 50 g/L λ-cyhalothrin), and fungicide (first: 1 L/ha Amistar Extra, 200 g/L azoxystrobin, and 80 g/L cyproconazole; second: 1 L/ha Cherokee, containing 50 g/L cyproconazole, 62 g/L propiconazole, and 375 g/L chloretalonil) each year. The temperature was typical for Hungary during the 2018–2019 season but with more colder days during winter and lower minimum temperatures in the last 100 days before harvest than in the 2013–2014 season. The quantity of precipitation was slightly below the average in both years, with a higher amount falling in the last 100 days before harvest [26] (Table S2).

The entire Mv Toborzó/Tommi RIL population was sown in field experiments in Martonvásár (under the conditions described earlier). The lines were sown in one replicate, while the two parents as controls were evenly distributed in the experiment in 6-6 replicates. At the end of the growing season, the compositional traits were measured from wholemeal by collecting, threshing, and grinding 6-6 ears per plot. The physical properties of the grains were determined by the seeds harvested with the combine, and the breadmaking quality traits were determined by the flour samples made from them.

### 2.3. Analysis of the Compositional Traits

The thousand-kernel weight (TKW) of the grains was measured (g/1000 grains) [27], and then 500 g of grains per sample were conditioned to a moisture content of 15.5% and ground with a Chopin CD1 Laboratory Mill (CHOPIN Technologies, Villeneuve-la-Garenne, France). The wholemeal samples were prepared with a Retsch MM400 mill. The crude protein content was determined by the Kjeldahl method according to ICC 105/2 (1995) [28] on a Kjeltec 1035 Analyzer (FOSS Tecator, Höganäs, Sweden). The gluten content was measured with a Perten Glutomatic 2200 [29] (ICC 137/1, 1995) (Perten, Hamburg, Germany). The starch content was measured by the NIR method with a Perten Inframatic 8611 instrument [30] (ICC 202, 1995). The total (TOT) and water-soluble (WE) pentosan contents were measured colorimetrically according to the Douglas method [31] (1981). The β-glucan content was determined according to AACC 32-23.01 [32] by enzymatic digestion and spectrophotometry. The measurements were performed in two replicates.

### 2.4. Analysis of Tocopherols and Tocotrienols

The tocopherol and tocotrienol contents of the wholemeal flour were determined by normal phase HPLC (NP-HPLC) with a fluorescence detector (FLD) after hot saponification of the lipids and extraction of the unsaponifiable lipids. In a modified method by Ryynänen et al. [33], a 0.5-g sample of flour was saponified with KOH in ethanol-water and under nitrogen at 100 °C for 25 min. Ascorbic acid was used as an antioxidant. The unsaponifiable lipids and tocols were extracted with the mixture of heptane:ethyl acetate (8:2). After washing the extract and evaporating the solvent, the residue was taken up in heptane and filtered through a Millex-LCR filter (0.45 μm, 13 mm) before analysis by NP-HPLC-FLD. Separation was performed on an Inertsil silica column (5 μm, 250 mm × 4.6 mm; Varian Chromapack, Middelburg, The Netherlands) with a silica precolumn (Guard-Pak Silica, Waters, Milford, MA, USA) and a heptane mobile phase containing 3% 1,4-dioxane. This was performed at a flow rate of 2 mL/min on a 30 °C column with an FLD detector at λ_ex_ = 292 nm and λ_em_ = 325 nm.

Commercial tocopherols (α-, β-, γ-, and δ-tocopherol; Merck, Darmstadt, Germany) were dissolved in ethanol (purity > 99.5% for spectrophotometric purposes), and this concentrate was used to identify and quantify the components. The concentration and purity of these solutions were checked by UV spectroscopy every 4 weeks, but work was carried out with a freshly diluted solution with heptane every 2 weeks. To record the calibration curve, 6 concentrations of each vitaminer were prepared in the range of 2–80 ng/injection. Quantification of the tocotrienols was determined using the appropriate tocopherol according to the AOCS method (1997) [34].

### 2.5. Analysis of Dough Properties

The dough properties (water uptake, dough development time, dough stability, and dough softening) were measured with a Brabender Farinograph according to ICC 115/1 (1995) [35] (Brabender, North Rhine-Westphalia, Duisburg, Germany). The gluten index (GI) was calculated according to ICC 155 (1995) [36]. The Zeleny sedimentation, which refers to the bread volume, was measured by the ICC 116/1 method (1997) [37] with a SediCom System [38]. The Falling Number was measured by the Perten Falling Number System 1500 (AACC 56-81B) [39].

### 2.6. Statistical Analysis

The least significant differences and correlations were calculated using Microsoft Excel. The construction of box and whisker plots and principal component analysis were carried out in Statistica 6.0. One-way ANOVA, Tukey’s post hoc test, hierarchical cluster analysis (with Ward’s method and the Euclidean distance) and linear mixed model analysis (using the restricted likelihood algorithm (REML)) were performed using SPSS Statistics 27.0 software (SPSS Inc., Chicago, IL, USA).

The linear mixed model analysis was based on the work of Virk et al. [40]. A total of 2 years were considered as different environments (E), and 2 technical replications were used in each environment for each genotype (G). In this model, replication was the random factor. A second model was used for the properties that showed a significant G × E interaction in the first model. The repeatability, genotypic variance, and variance of the G × E interaction were evaluated for each trait. Repeatability (broad sense heritability) was calculated as the ratio of genotypic to phenotypic variance.

Discriminant analysis was used to examine the shift of the population traits between years and their possible grouping.

## 3. Results

The contents and compositions of the tocols in the 230 Mv Toborzó/Tommi lines were determined in samples grown in 2 years (Table 1). Significant variation was found in the amounts of individual tocols, being from 1.38 to 10.05 μg/g d.m. for α-tocopherol, 1.01 to 4.79 μg/g d.m. for α-tocotrienol, 0.80 to 5.64 μg/g d.m. for β-tocopherol, and 8.55 to 28.94 μg/g d.m. for β-tocotrienol (Table 1). The average amounts were 5.58 μg/g d.m. for the α-tocopherols, 2.53 μg/g d.m. for the α-tocotrienols, 3.07 μg/g d.m. for the β-tocopherols, and 16.12 μg/g d.m. for the β-tocotrienols. The total tocol content ranged from 13.69 to 45.18 μg/g d.m. with an average of 27.53 μg/g d.m. The γ-tocopherols were present in trace amounts in the wheat lines. The 1000-kernel weight (TKW) ranged from 25.97 to 58.52 g with an average of 43.47 g (Table 1 and Table S3). The highest total tocol content was found in lines 3, 12, 32, and 39, while the highest amount of α-tocopherol was present in lines 4, 12, 54, and 75 (Table S3).

A comparison of the contents of the tocols in the RILs and parental cultivars (Mv Toborzó and Tommi) showed transgressive segregation (Figure S1), with Mv Toborzó and Tommi having 21.65 and 33.37 μg/g d.m. total tocol contents, 5.24 and 6.08 μg/g d.m. α-tocopherol contents, 11.11 and 20.52 μg/g d.m. β-tocotrienol contents, 2.27 and 3.08 μg/g d.m. α-tocotrienol contents, and 2.79 and 3.54 μg/g d.m. β-tocopherol contents, respectively.

The thousand-kernel weight showed significant negative correlations with the total tocol content (r = −0.4331, r_5%_ =0.1236) and the contents of β-tocopherol (r = −0.3946, r_5%_ = 0.1236) and β-tocotrienol (r = −0.5062, r_5%_ = 0.1236, r_0_._1%_ = 0.2061). This shows that the larger kernels contained relatively less tocols, β-tocopherol, and β-tocotrienol, which is consistent with their concentrations in the outer layers of the seed and the embryos.

**Figure 1:**
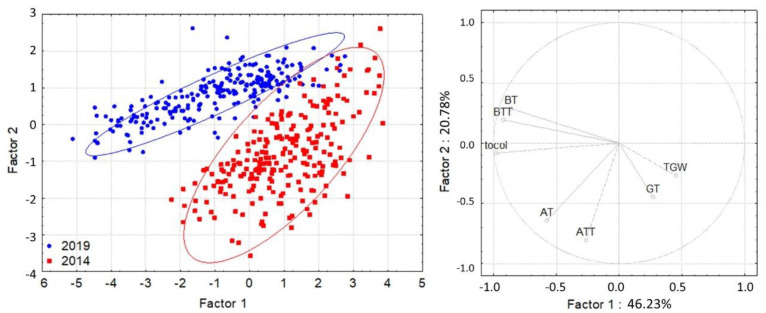
Principal component analysis based on the content and composition of tocols in the grain. AT = α-tocopherol, ATT = α-tocotrienol, BT = β-tocopherol, BTT = β-tocotrienol, GT = γ-tocopherol, tocol = total tocol content, and TKW = thousand-kernel weight (where the most determinant traits contributing to Factor 1 were BT (−0.8607), BTT (−0.9239), and tocol (−0.9814), while it was AT (−0.6417) and ATT (−0.8032) for Factor 2).

The size distributions of the kernels were similar in the 2 years of the study. However, the β-tocopherol, β-tocotrienol, and total tocol contents were higher in 2019 than in 2014 (Figure 1).

The total contents and compositions of the tocols were also significantly affected by the genotype (G), environment (E), and the interaction of these two factors (G × E) (Table 2).

**Figure 2:**
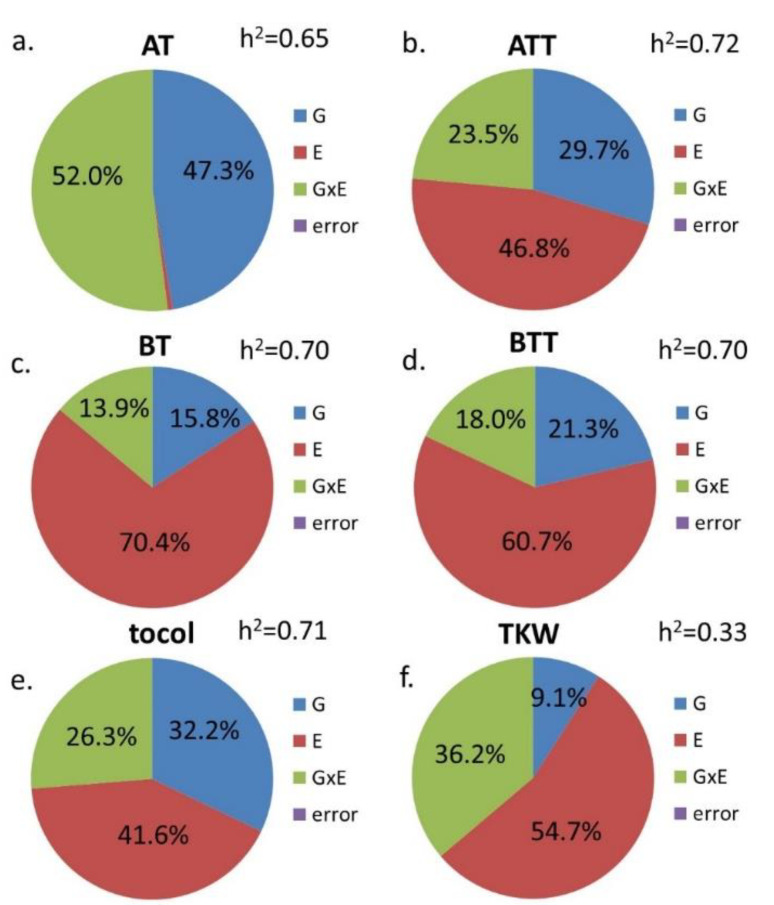
Pie diagrams of the relative contribution of genotype (G), environment (E, 2 years), and genotype × environment interaction (G × E) to the total sum of squares for the tocol content and composition of bread wheat, where (**a**) AT = α-tocopherol, (**b**) ATT = α-tocotrienol, (**c**) BT = β-tocopherol, (**d**) BTT = β-tocotrienol, (**e**) tocol = total tocol content, and (**f**) TKW = thousand-kernel weight (Martonvásár, 2014 and 2019, *n* = 230).

The genotype (G) determined 47.3% of the total variance in the amount of α-tocopherol and 29.7% for α-tocotrienol, 15.8% for β-tocopherol, 21.3% for β-tocotrienol, and 32.2% for the total tocol content (Figure 2). The dominant role of the genotype was confirmed by the broad sense heritability values, which were 0.65 for α-tocopherol and about 0.7 for the other tocol components. A strong effect from the environment (E) was also shown for α-tocotrienol, β-tocopherol, β-tocotrienol, and the total tocol content, as was a significant effect from G × E on α-tocopherol (Figure 2). The variance in kernel weight was determined less by the genotype (9.1%) with a low heritability (0.33), while the effects of E and G × E were more significant (54.7 and 36.2%, respectively).

**Figure 3:**
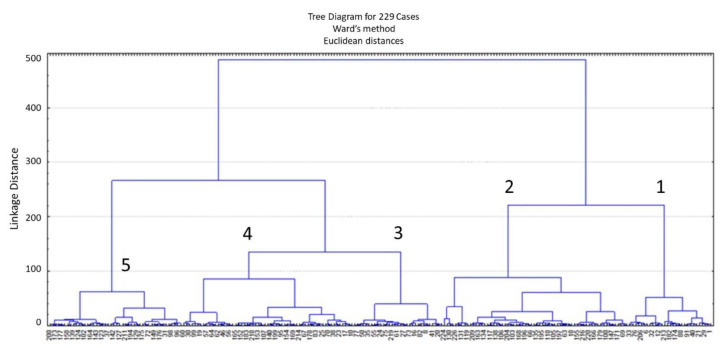
Hierarchical cluster analysis based on the tocol content and composition (see also a more detailed figure in Figure S2).

**Figure 4:**
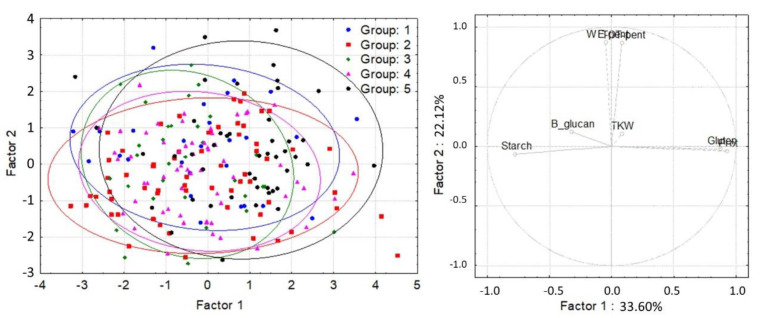
Principal component analysis based on the compositional quality traits of wheat and grouped by the groups formed by the hierarchical cluster analysis. B-glucan = β-glucan content, Gluten = gluten content, Prot = protein content, Starch = starch content, TKW = thousand-kernel weight, TOT-pent = total pentosan content, and WE-pent = water-extractable pentosan content (where the most determinant traits contributing to Factor 1 were Prot (0.9279), Gluten (0.8768), and Starch (−0.7757), while they were TOT-pent (0.8686) and WE-pent (0.8728) for Factor 2).

**Figure 5:**
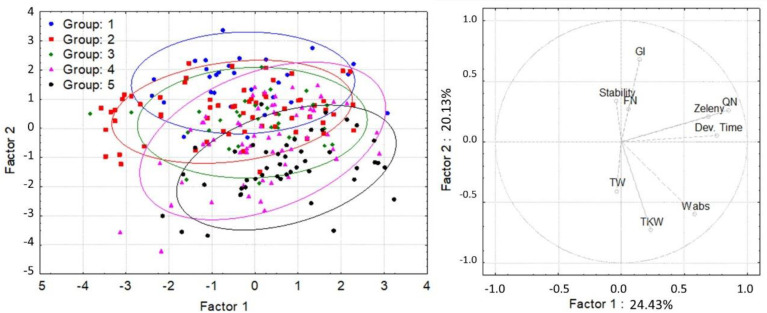
Principal component analysis based on the breadmaking quality traits of wheat and grouped by the groups formed by the hierarchical cluster analysis. Dev Time = dough development time, FN = falling number, GI = gluten index, QN = farinograph quality number, Stability = dough stability time, Zeleny = Zeleny sedimentation volume, TKW = thousand-kernel weight, TW = test weight, and Wabs = water absorption (where the most determinant traits contributing to Factor 1 were Zeleny (0.6913), Dev Time (0.7594), and QN (0.8517), while they were GI (0.6779), Wabs (−0.5973), and TKW (−0.7269) for Factor 2).

Hierarchical cluster analysis based on the tocol content and composition yielded five main groups (Figure 3 and Figure S2). The compositional quality traits and the breadmaking quality of these five main groups were compared by principal component analysis (Figure 4 and Figure 5). The compositions of the lines in the five groups were very similar with the groups overlapping (Figure 4). However, some lines in Group 5 showed significantly higher-than-average contents of water-extractable pentosans (lines 37, 123, 177, and 188 with 13.4–13.8 mg/g WE-pentosan content). These lines also had high water absorption (63.2–69.6%). Significant differences were observed in the breadmaking quality of the groups, with the lines in Group 1 having a significantly higher gluten index but lower water absorption and kernel weight than those in Group 5 (the lines with the highest contents were 1, 12, 32, and 195 with 99.1–99.8 GI, 56.3–58.8% Wabs, and 35.5–41.0 g TKW ranges, while the maximum values in the population were 99.8, 70.9%, and 58.5 g, respectively) (data shown with molecular studies of the same population) (Figure 5).

According to the Tukey test (Table 3), Group 3 contained the lines with the highest average content of both tocopherols (α and β), while the lines of Group 1 were the richest in both tocotrienols. The average total tocol contents of these two groups (1 and 3) were the highest and did not differ significantly from each other. The average contents of the total and individual tocols were significantly lower in Group 5 than those in the other groups but did not differ significantly from Group 2 in terms of the tocopherol contents.

The Tukey test showed significant differences between the five groups for all compositional and quality traits, except the total pentosan content, dough stability, dough development time, Farinograph quality number, and Zeleny sedimentation (Table 3). Although the first two factors of PCA could not differentiate the five groups based on their compositional quality traits, the Tukey test showed that the average protein content of Group 5 was significantly higher than the protein contents of the other four groups. The WE-pentosan content of Group 1 was significantly higher than those of Groups 2 and 4. The β-glucan content of Group 1 was significantly lower than those of Groups 2, 4, and 5. The starch contents of Groups 3 and 4 were significantly higher than those of Groups 1 and 2. Finally, Group 5 had the highest gluten content, while Group 1 had the lowest.

Of the parameters determining the dough quality, the gluten indices of Groups 1 and 3 were significantly higher for Groups 1 and 3 than for Group 5, while the average water absorption was highest for Group 5 and lowest for Group 1. The falling number was significantly higher for Groups 1 and 3 than for the other three groups. Of the physical traits of the kernels, the TKWs of the five groups differed significantly from each other (in increasing order from Group 1 to Group 5), while the test weights of Groups 1 and 2 were significantly lower than those of Groups 3, 4, and 5.

## 4. Discussion

Most reported studies on tocols in wheat have included only a few wheat genotypes (8), with average total tocol values of 27.8–37.1 μg/g determined by Barnes [41], Zielisnki et al. [42], and Ruibal-Mendieta et al. [43]. The average total tocol content in our lines (27.5 μg/g) agreed with these values. However, Panfili et al. [6] reported a very high total tocol content of 74.3 μg/g. The Healthgrain diversity screen analyzed 130 winter wheat genotypes, reporting an average total tocol content of 49.9 μg/g with a range of 27.6–79.7 μg/g. The total tocol content in our RILs was between 13.7 and 45.2 μg/g, representing a threefold variation, which was only slightly higher than the fold variation in the Healthgrain lines (2.9-fold). In general, β-tocotrienols (>50%) and α-tocopherols (>20%) were the major tocols, followed by α-tocotrienol and β-tocopherol, with α-tocopherol having the greatest vitamin E activity. In the Healthgrain program, the α-tocopherol content varied between 6.4 and 19.9 μg/g, which represented 12.2–40.8% of the total tocol content, representing a threefold variation [25,44]. Although the average values for the tocols were lower in the present study, the range of values for α-tocopherol was greater, being between 1.4 and 10 μg/g and representing 10–22% of the total tocol content with variation of sevenfold. Moore et al. [45] reported 3.4–10 μg/g α-tocopherol in wheat, representing a threefold variation. The ratio of the total tocotrienols (α and β) to the total tocols was ≥62.5% in the Healthgrain samples [44] and was reported to be 70% by Zielinski et al. [42] and 66–77% by Panfili et al. [6]. The ratio of 70–75% reported here is therefore consistent with these studies. It is of interest that Hidalgo et al. [46] and Lampi et al. [44] analyzed the same two varieties (Mieti and Sagittario) grown on sites in Italy and Hungary and reported significantly different values. The total tocol contents were 63.7 and 55.5 μg/g for Mieti and Sagittario in Italy, while they were 48.5 and 47.4 μg/g in Hungary, respectively. From these, the ratio of total tocotrienols were 67.1%, 61.5%, 60.5% and 49.5%, respectively. All these datasets show that although the mean concentrations determined in this study were lower than in previous reports, the ratios of the different tocol components and the fold ranges in the concentrations were similar. These differences are not surprising, as the concentrations of phytochemicals are highly dependent on the species, the genotypes, the geographical origin of the genotypes, the environmental conditions (growing site, year, field management, etc.), and the analytical methods used.

In relation to the environmental effects, Lampi et al. [10] studied the tocol compositions in 26 wheat genotypes grown at one site for 3 years and at three other sites for 1 year. In general, both genetic and environmental factors had strong effects on the tocol contents of the wheat genotypes. As the growing sites and years differed widely (with locations in four European countries), greater effects were observed from the environment than in earlier studies. In addition, some genotypes were more sensitive to environmental effects, whereas others were relatively stable (Claire, Cadenza, Lynx, Atlas 66, and Disponent) [10]. These genotypes were considered to be potential candidates for breeding wheat cultivars with high stable tocol contents. In our study, the effect of the environment was also significant and determined 41.6% of the total variance of the total tocols. However, the amount of α-tocopherols, which exhibit activity as vitamin A, was more strongly determined by the genotype, which accounted for 47.3% of the total variance, while the effect of the environment was low (0.7%). This finding supports the suggestion that tocols, and particularly α-tocopherol, are suitable targets for enhancement by wheat breeding.

Tocols are concentrated in the outer layers and germ (181–320 μg/g d.m.) of the grain [41]. When the grain is milled, these tissues form the bran fraction, which contains 76–95 μg/g d.m. compared with 17–44 μg/g d.m. in the starchy endosperm (white flour) [47]. The germ contains mainly α- and β-tocopherols (104–221 μg/g d.m. and 67–86 μg/g d.m., respectively) [1]. Tocotrienols are concentrated in the pericarp, testa, and the aleurone layer at 11–15 μg/g d.m. and 44–56 μg/g d.m. for α- and β-tocotrienols, respectively, accounting for 85% of the total tocotrienol content of the grain. They also present in significant amounts in the starchy endosperm, being 1.0–2.5 μg/g d.m. and 11–17 μg/g d.m. for α- and β-tocotrienols, respectively, accounting for about 15% of the total grain content [48], but they are not present in significant amounts in the germ [1]. High proportions of β-tocotrienols were also present in the wheat RIL population. The amounts of α- and β-tocopherols in the starchy endosperm (white flour) ranged between 2.0 and 16.0 and 1.0 and 8.0 μg/g d.m., compared with 13 and 16 and 6.6 and 8.0 μg/g d.m., respectively, in the bran. The tocols were also concentrated in the bran fractions of the RIL population, with higher contents of tocols, β-tocopherol, and β-tocotrienol in small seeds. The γ and δ forms of tocols are present in significant amounts in barley, while small amounts of all forms of tocols are present in oats. Very small amounts of γ-tocopherol were also present in the RIL population (0.23 μg/g d.m. on average). Although cereals are moderate sources of tocols compared with oils, the vitamin E activity of oil extracted from wheat germ has been found to be higher than that of oils from most other sources [49].

The current work on improving the nutrient compositions of crops to reduce global deficiencies has focused on three micronutrients: iron (Fe), zinc (Zn), and vitamin A (HarvestPlus program, harvestplus.org). This has resulted in new, value-added, vitamin A-rich sweet potatoes, yellow manioc, orange corn, iron-rich beans and Indian millet, and zinc-rich rice and wheat, which have been produced and tested in 40 countries [50,51]. The biofortified types of yellow cassava, orange corn, and potatoes can provide 100% of the nutritional requirements, but cultural factors can limit the acceptance of the new types of foods, which differ in taste, texture, and colour from traditional types [52]. In addition to B vitamins, wheat also contains a significant amount of vitamin E. In the case of vitamins, it should be borne in mind that the bioactivity, digestibility, and bioavailability of different vitamins may be different. There has been less interest in increasing the vitamin E content of cereals [53], although a fourfold increase in the amount of α-tocopherol has been reported in maize [54], while a threefold increase in α-tocotrienols was achieved in rice [55] compared with a control.

Previous studies showed that the contents of tocols, sterols, and alkylresorcinols in wheat are highly heritable (above 50%), while the contents of B vitamins (B1, B2, B3, and B6), methyl donors (choline and betaine), and phenolic acids have low heritability, with high environmental or G × E impact [7,56]. In our study, the genotype determined 16–47% of the total variance, depending on the tocol component. The broad sense heritability of the components ranged from 0.65 to 0.72, showing that they are highly heritable and hence appropriate targets for selection in plant breeding.

## 5. Conclusions

We have shown wide variation (threefold) in the total content of tocols in a biparental wheat RIL population, with α-tocopherol (vitamin E) being the major component, and identified lines with high contents of total tocols (3, 12, 32, and 39) and α-tocopherol (4, 12, 54, and 75). We have also shown that tocols have high heritability (0.7 for the total tocols and 0.65 for α-tocopherol), indicating that they should be amenable for selection by wheat breeders. The groups of lines with the highest average contents of tocopherols and tocotrienols (Group 3 and Group 1, respectively) had the lowest average protein content but the highest gluten index and Falling Number. Furthermore, Group 3 also had a significantly higher average starch content, while Group 1 had a significantly higher WE-pentosan content and lower TKW than all the other groups. The lines in the group with the lowest average tocol contents (Group 5) had the highest average contents of protein, gluten, and β-glucan, while their water absorption and thousand-kernel weights were also the highest. All this means that the main component that presumably affects the water uptake of the lines is the protein in the large-kernel, low-tocol Group 5, the pentosan in small-kernel, high-tocotrienol Group 1, and starch in the high-tocopherol Group 3. However, tocols are sensitive to oxidation during grain processing, and care will be required to minimize losses if improved genotypes are produced to increase the contribution of wheat to vitamin E intake.

## Figures and Tables

**Table 1:** Mean, standard deviation, and minimum and maximum values of tocopherols and tocotrienols in 230 lines of Mv Toborzó/Tommi population in 2 years (2014 and 2019).

Descriptives	Year	N	Mean	Std. Dev.	Std. Error	95% Confidence Interval for Mean		Min.	Max.
			μg/g			Lower Bound	Upper Bound	μg/g	μg/g
AT	2014	458	5.67	1.82	0.09	5.51	5.84	1.38	10.05
	2019	458	5.48	1.04	0.05	5.38	5.57	3.30	8.39
	Total	916	5.58	1.49	0.05	5.48	5.67	1.38	10.05
ATT	2014	458	2.91	0.57	0.03	2.85	2.96	1.15	4.79
	2019	458	2.16	0.56	0.03	2.11	2.21	1.01	3.88
	Total	916	2.53	0.67	0.02	2.49	2.58	1.01	4.79
BT	2014	458	2.40	0.45	0.02	2.36	2.45	0.80	3.76
	2019	458	3.75	0.75	0.03	3.68	3.81	1.84	5.64
	Total	916	3.07	0.91	0.03	3.02	3.13	0.80	5.64
BTT	2014	458	13.19	2.25	0.11	12.97	13.40	8.55	19.12
	2019	458	19.05	4.13	0.19	18.67	19.43	10.51	28.94
	Total	916	16.12	4.43	0.15	15.83	16.41	8.55	28.94
GT	2014	458	0.31	0.39	0.02	0.27	0.34	0.00	1.39
	2019	458	0.15	0.07	0.00	0.14	0.15	0.06	0.43
	Total	916	0.23	0.29	0.01	0.21	0.25	0.00	1.39
Tocol	2014	458	24.48	4.12	0.20	24.09	24.87	13.69	34.87
	2019	458	30.61	5.93	0.28	30.04	31.13	17.82	45.18
	Total	916	27.53	5.94	0.20	27.14	27.92	13.69	45.18
TKW	2014	458	46.47	5.93	0.30	45.87	47.07	25.97	58.52
	2019	458	40.44	5.39	0.34	39.84	41.04	26.90	53.47
	Total	916	43.47	6.40	0.23	43.02	43.92	25.97	58.52

AT = α-tocopherol, ATT = α-tocotrienol, BT = β-tocopherol, BTT = β-tocotrienol, GT = γ-tocopherol, tocol = total tocol content, and TKW = thousand-kernel weight. Based on data in Table S3.

**Table 2:** Effect of the genotype, the environment, and their interaction with the tocol content and composition of the Mv Toborzó/Tommi lines (2014 and 2019).

	G	E	G × E
AT	***	***	***
ATT	***	***	***
BT	***	***	***
BTT	***	***	***
GT	***	***	***
tocol	***	***	***
TKW	***	***	n.s.

*n* = 230, AT—α-tocopherol, ATT—α-tocotrienol, BT—β-tocopherol, BTT—β-tocotrienol, GT—γ-tocopherol, tocol—total tocol content, TKW—thousand kernel weight. *** significant at the 0.001 probability level. n.s.—not significant.

**Table 3:** Differences among the means of the five groups of the lines, determined by the hierarchical cluster analysis and evaluated by Tukey test.

Group	1	2	3	4	5
N	29	64	31	59	46
α-tocopherol content (μg/g)	6.38 ^bc^	4.87 ^a^	6.89 ^c^	5.85 ^b^	4.82 ^a^
α-tocotrienol content (μg/g)	3.29 ^a^	2.42 ^b^	2.87 ^b^	2.48 ^c^	2.06 ^d^
β-tocopherol content (μg/g)	3.47 ^c^	2.85 ^a^	3.60 ^c^	3.17 ^b^	2.66 ^a^
β-tocotrienol content (μg/g)	20.40 ^d^	15.22 ^b^	19.17 ^c^	16.02 ^b^	12.76 ^a^
γ-tocopherol content (μg/g)	0.18 ^ab^	0.28 ^c^	0.12 ^a^	0.22 ^ab^	0.26 ^c^
Total tocol content (μg/g)	33.72 ^d^	25.63 ^b^	32.65 ^d^	27.74 ^c^	22.56 ^a^
TOT-pentosan content (mg/g)	46.82 ^a^	44.82 ^a^	44.67 ^a^	44.58 ^a^	46.39 ^a^
WE-pentosan content (mg/g)	10.25 ^b^	8.87 ^a^	9.55 ^ab^	9.05 ^a^	9.78 ^ab^
B-glucan content (mg/g)	6.44 ^a^	6.98 ^b^	6.90 ^ab^	7.02 ^b^	6.99 ^b^
Protein content (%)	14.14 ^a^	14.31 ^a^	14.06 ^a^	14.38 ^a^	14.98 ^b^
Starch content (%)	55.11 ^a^	55.42 ^a^	56.11 ^b^	56.16 ^b^	55.64 ^ab^
Gluten content (%)	31.73 ^a^	32.70 ^ab^	33.32 ^ab^	34.18 ^bc^	36.43 ^c^
Gluten Index (GI)	89.90 ^b^	86.40 ^ab^	87.69 ^b^	85.85 ^ab^	80.52 ^a^
Falling Number (FN) (s)	408.85 ^b^	319.59 ^a^	403.89 ^b^	345.94 ^a^	295.83 ^a^
Zeleny sedimentation (ml)	45.20 ^a^	41.50 ^a^	44.58 ^a^	44.53 ^a^	44.20 ^a^
Dough development time (Dev Time) (min)	9.37 ^a^	9.74 ^a^	9.45 ^a^	10.21 ^a^	11.45 ^a^
Dough Stability (min)	12.86 ^a^	13.39 ^a^	12.14 ^a^	13.23 ^a^	13.40 ^a^
Water absorption (Wabs) (%)	60.05 ^a^	61.25 ^ab^	61.03 ^ab^	62.12 ^b^	64.41 ^c^
Quality number (QN)	88.21 ^a^	84.33 ^a^	86.98 ^a^	86.71 ^a^	88.82 ^a^
Thousand-kernel weight (TKW) (g)	36.78 ^a^	39.46 ^b^	45.49 ^c^	48.55 ^d^	50.82 ^e^
Test weight (TW) (kg/100 L)	70.62 ^a^	72.52 ^a^	75.48 ^b^	76.56 ^b^	75.94 ^b^

The mean difference is significant at the 0.05 level if the letter is different.

## Data Availability

The data presented are available in the article and its supplementary material.

## Supplementary Materials

The following supporting information can be downloaded at https://www.mdpi.com/article/10.3390/foods11091343/s1. Figure S1: Frequency distribution of the tocol content in the RIL population ((a) total tocol, (b) α-tocopherol, (c) β-tocotrienol, (d) α-tocotrienol, and (e) β-tocopherol); Figure S2: Hierarchical cluster analysis based on the tocol content and composition of the individual lines; Table S1: Growing and environmental conditions in Hungary; Table S2: Meteorological conditions in Hungary; Table S3: Average of the compositional traits in the individual lines (2014, 2019).
