# Supplementary material for: Variation in the Content and Composition of Tocols in a Wheat Population
Source: Foods. 2022 May 5;11(9):1343. doi: 10.3390/foods11091343 (PMC9105132; doi:10.3390/foods11091343)
Supplement: Supplementary file 1 [file foods-11-01343-s001.zip › Supplemetary Tables.pdf]

Table S1. Growing and environmental conditions in Hungary.

Growing conditions		2013/2014	2018/2019
**Location**	geographic coordinates	47°3'N, 18°8'E	47º18'N, 18º47'E
	altitude	115 m	115 m
**Growing parameters**	previous crop:	oilseed radish	oilseed radish
	sowing density	550 seeds/m2	550 seeds/m^2
**Soil parameters**	soil type	chernozem	chernozem
	pH (KCl)	7.25	7.25
	humus (m/m%)	2.8	2.8
	P_2O_5 (mg/kg)	210	210
	K_2O (mg/kg)	210	210
	yearly average N input through NPK combined fertilizer (active ingredient, kg/ha)	120	120

Table S2. Meteorological conditions in Hungary.

		2013/2014	2018/2019
**full season**	Growing period (days)	268	279
	Cumulative precipitation (mm)	348.9	365.6
	Mean temperature (°C)	10.44	9.3
	Absolute min temperature (°C)	-11.8	-14.4
	Absolute max temperature (°C)	33.8	36.0
**last 100 days**	Cum. precipitation in the last 100 days before harvest (mm)	202.6	225.0
	Mean temperature in the last 100 days (°C)	18.33	17.1
	Absolute min temp in the last 100 days (°C)	2.6	-0.7
	Absolute max temp in the last 100 days (°C)	33.8	36.0
**abs. min-max**	No of days with Tmin ≤ 0 °C	71	90
	No of days with Tmin ≤ -10 °C	1	6
	No of days with Tmax ≥ 25 °C	54	42
	No of days with Tmax ≥ 30 °C	18	16
	No of days with Tmax ≥ 35 °C	0	1

Table S3. Average of the compositional traits in the individual lines (2014, 2019).

Line	Year	AT	ATT	BT	BTT	GT	total tocol	TKW	Group
		ug/g	ug/g	ug/g	ug/g	ug/g	ug/g	g	
**MV-TOBORZO**	**2014**	5,50	2,53	2,23	8,04	0,38	18,67	55,96	5
**MV-TOBORZO**	**2019**	4,98	2,00	3,35	14,18	0,12	24,63	49,10	5
**TOMMI**	**2014**	6,12	3,64	2,75	17,82	0,10	30,42	42,03	1
**TOMMI**	**2019**	6,04	2,52	4,32	23,22	0,20	36,31	31,90	1
**TT1**	**2019**	6,17	3,51	4,45	25,13	0,18	39,43	34,88	1
**TT2**	**2019**	7,32	2,71	4,80	22,09	0,16	37,08	35,87	1
**TT3**	**2019**	6,81	2,70	5,15	28,61	0,10	43,38	41,17	1
**TT4**	**2019**	7,51	2,53	5,23	21,57	0,12	36,97	42,02	3
**TT5**	**2019**	6,34	2,57	4,74	22,18	0,16	35,99	41,44	3
**TT6**	**2019**	5,48	3,10	3,29	28,94	0,11	40,92	38,13	1
**TT7**	**2019**	6,59	2,83	4,83	25,34	0,14	39,74	42,65	3
**TT8**	**2019**	6,80	2,51	4,53	24,79	0,14	38,76	44,70	3
**TT9**	**2019**	5,76	2,92	3,82	22,24	0,14	34,88	38,07	2
**TT10**	**2019**	6,01	1,88	4,30	19,52	0,13	31,85	47,68	4
**TT11**	**2019**	7,18	1,85	5,15	18,02	0,11	32,32	45,44	4
**TT12**	**2019**	7,58	3,03	4,97	27,34	0,10	43,03	42,22	1
**TT13**	**2019**	5,87	2,48	3,83	23,03	0,09	35,29	46,91	4
**TT14**	**2019**	5,87	2,22	3,73	15,04	0,10	26,95	53,47	5
**TT15**	**2019**	7,33	2,99	4,96	24,02	0,10	39,41	35,23	1
**TT16**	**2019**	6,08	3,03	4,39	26,29	0,11	39,89	42,51	3
**TT17**	**2019**	5,52	2,49	3,59	19,17	0,09	30,86	41,95	4
**TT18**	**2019**	5,91	2,39	4,60	18,73	0,14	31,78	38,25	2
**TT19**	**2019**	5,72	1,70	3,71	18,36	0,11	29,60	47,87	4
**TT20**	**2019**	6,68	2,34	5,07	20,71	0,13	34,94	45,88	3
**TT21**	**2019**	6,94	3,34	4,59	25,01	0,12	40,01	30,70	1
**TT22**	**2019**	6,48	2,56	3,75	22,77	0,12	35,69	40,27	3
**TT23**	**2019**	5,36	2,06	3,66	19,59	0,13	30,81	45,80	4
**TT24**	**2019**	6,58	2,88	3,83	21,23	0,09	34,61	42,95	3
**TT25**	**2019**	6,33	2,27	4,14	19,00	0,07	31,81	40,89	4
**TT26**	**2019**	6,45	2,63	4,71	26,14	0,10	40,02	43,89	3
**TT27**	**2019**	5,51	2,26	4,22	21,16	0,09	33,24	42,06	3
**TT28**	**2019**	5,60	2,19	3,92	21,30	0,09	33,10	46,09	3
**TT29**	**2019**	6,61	3,35	4,16	26,87	0,14	41,14	34,99	1
**TT30**	**2019**	6,63	2,42	4,08	18,25	0,15	31,54	53,26	4
**TT31**	**2019**	5,09	2,21	3,23	16,71	0,17	27,41	46,68	5
**TT32**	**2019**	7,68	3,25	4,89	26,51	0,15	42,49	38,00	1
**TT33**	**2019**	7,40	3,48	5,33	25,26	0,11	41,58	36,08	1
**TT34**	**2019**	7,31	2,37	3,66	17,09	0,15	30,57	46,66	5
**TT35**	**2019**	5,26	2,76	3,46	26,31	0,12	37,90	38,30	3
**TT36**	**2019**	5,36	2,50	3,87	21,31	0,11	33,15	40,94	4
**TT37**	**2019**	5,01	2,12	3,09	16,80	0,08	27,09	49,04	5

**TT38**	**2019**	4,85	2,01	3,16	21,72	0,07	31,81	41,48	4
**TT39**	**2019**	6,57	2,97	5,29	26,74	0,14	41,70	42,61	1
**TT40**	**2019**	6,53	3,18	5,04	25,34	0,14	40,23	36,01	1
**TT41**	**2019**	6,56	2,47	4,96	25,66	0,12	39,76	43,30	3
**TT42**	**2019**	5,82	3,88	3,73	27,03	0,19	40,65	34,08	2
**TT43**	**2019**	7,66	2,02	4,90	18,22	0,15	32,95	47,84	4
**TT44**	**2019**	6,39	2,33	4,41	21,21	0,12	34,46	45,52	4
**TT45**	**2019**	6,08	2,93	4,05	25,02	0,14	38,23	40,51	1
**TT46**	**2019**	6,71	2,99	4,26	25,08	0,09	39,14	44,89	4
**TT47**	**2019**	5,26	2,68	3,95	22,31	0,11	34,32	46,18	4
**TT48**	**2019**	6,08	3,22	3,88	28,85	0,11	42,13	38,86	1
**TT49**	**2019**	6,73	3,00	4,82	27,96	0,14	42,64	35,16	1
**TT50**	**2019**	5,76	2,04	4,42	24,21	0,13	36,57	39,34	3
**TT51**	**2019**	5,77	3,23	4,21	27,21	0,21	40,63	33,42	1
**TT52**	**2019**	6,99	2,40	4,56	17,73	0,10	31,77	47,89	4
**TT53**	**2019**	5,70	2,41	3,70	20,61	0,14	32,57	35,14	2
**TT54**	**2019**	8,05	2,29	5,09	25,45	0,12	41,00	44,59	3
**TT55**	**2019**	6,29	2,12	4,13	23,46	0,12	36,12	43,51	3
**TT56**	**2019**	6,05	2,36	4,09	23,48	0,11	36,09	45,62	4
**TT57**	**2019**	5,53	2,03	4,21	20,73	0,09	32,60	47,94	4
**TT58**	**2019**	5,65	2,55	4,14	24,19	0,09	36,62	39,14	3
**TT59**	**2019**	5,21	2,10	4,04	17,23	0,10	28,67	44,58	5
**TT60**	**2019**	6,08	2,53	4,19	21,34	0,11	34,25	48,68	4
**TT61**	**2019**	6,63	2,45	4,23	21,81	0,10	35,22	40,17	3
**TT62**	**2019**	6,58	2,28	4,95	22,70	0,11	36,62	40,91	4
**TT63**	**2019**	5,52	2,71	3,72	19,93	0,12	31,99	34,76	2
**TT64**	**2019**	6,50	2,10	4,46	20,51	0,11	33,68	47,18	4
**TT65**	**2019**	6,17	1,86	4,90	16,82	0,11	29,87	41,13	4
**TT66**	**2019**	6,24	1,96	4,29	18,08	0,13	30,70	38,99	2
**TT67**	**2019**	5,27	1,86	3,37	21,46	0,09	32,05	40,61	4
**TT68**	**2019**	5,37	1,89	3,83	18,72	0,11	29,93	42,06	4
**TT69**	**2019**	6,50	2,50	4,10	24,76	0,16	38,03	36,90	2
**TT70**	**2019**	7,69	2,36	5,11	15,94	0,17	31,28	45,79	4
**TT71**	**2019**	5,82	2,16	3,59	22,32	0,10	33,98	44,83	4
**TT72**	**2019**	6,40	1,81	4,87	15,20	0,19	28,48	45,61	5
**TT73**	**2019**	8,27	3,00	5,62	28,17	0,12	45,18	40,56	3
**TT74**	**2019**	6,57	3,47	4,64	23,82	0,16	38,66	41,21	4
**TT75**	**2019**	8,39	2,32	5,51	22,71	0,14	39,07	38,57	3
**TT76**	**2019**	7,50	3,34	4,75	26,91	0,13	42,62	33,80	1
**TT77**	**2019**	7,29	2,42	5,29	25,31	0,10	40,40	43,34	3
**TT78**	**2019**	5,09	2,85	3,89	27,95	0,08	39,86	40,20	1
**TT79**	**2019**	7,07	2,67	5,64	21,79	0,13	37,30	39,90	3
**TT80**	**2019**	7,01	2,72	4,61	24,60	0,09	39,03	40,11	3
**TT81**	**2019**	6,59	2,24	4,09	22,82	0,08	35,83	39,36	3
**TT82**	**2019**	7,30	3,87	5,19	25,57	0,11	42,05	37,61	3
**TT83**	**2019**	6,53	2,54	4,63	19,45	0,15	33,29	37,68	4

**TT84**	**2019**	7,33	2,64	4,56	22,53	0,13	37,20	39,21	3
**TT85**	**2019**	7,16	2,13	4,34	21,32	0,10	35,05	43,78	3
**TT86**	**2019**	5,24	1,75	4,16	16,86	0,11	28,12	37,82	2
**TT87**	**2019**	6,58	2,99	4,53	21,64	0,13	35,86	33,99	1
**TT88**	**2019**	7,48	2,81	4,76	21,33	0,15	36,53	31,27	1
**TT89**	**2019**	6,12	2,86	4,38	23,58	0,08	37,03	44,19	4
**TT90**	**2019**	6,41	2,32	4,41	21,36	0,19	34,70	47,27	4
**TT91**	**2019**	6,58	3,45	4,23	24,35	0,14	38,75	32,32	1
**TT92**	**2019**	6,10	2,46	4,03	21,68	0,13	34,40	38,71	4
**TT93**	**2019**	6,55	3,50	4,92	23,42	0,19	38,58	38,81	3
**TT94**	**2019**	7,26	2,39	4,81	21,02	0,18	35,66	44,97	4
**TT95**	**2019**	5,71	2,52	3,48	20,20	0,10	32,02	39,81	4
**TT96**	**2019**	5,59	1,86	3,95	17,19	0,14	28,71	46,27	5
**TT97**	**2019**	6,54	2,42	4,69	21,50	0,15	35,30	39,78	3
**TT98**	**2019**	5,36	2,56	3,29	18,67	0,06	29,93	45,35	5
**TT99**	**2019**	4,07	2,18	2,94	20,32	0,11	29,62	44,94	4
**TT100**	**2019**	5,93	2,08	4,28	17,99	0,13	30,42	36,25	2
**TT101**	**2019**	5,88	2,49	3,92	16,30	0,43	29,02	33,51	2
**TT102**	**2019**	4,68	1,51	3,02	15,24	0,35	24,80	44,44	5
**TT103**	**2019**	4,80	1,67	3,13	15,57	0,33	25,50	39,89	2
**TT104**	**2019**	4,65	1,75	3,40	15,09	0,37	25,27	31,85	2
**TT105**	**2019**	3,90	1,88	2,92	16,32	0,28	25,32	37,53	2
**TT106**	**2019**	3,93	1,88	2,54	16,99	0,22	25,57	36,17	2
**TT107**	**2019**	4,51	2,11	3,06	18,24	0,33	28,26	40,11	4
**TT108**	**2019**	4,93	1,98	3,55	16,90	0,38	27,74	44,45	4
**TT109**	**2019**	4,56	1,41	2,73	12,58	0,29	21,57	47,34	5
**TT110**	**2019**	4,63	1,76	2,87	15,56	0,30	25,12	45,46	4
**TT111**	**2019**	5,45	1,84	3,86	18,19	0,41	29,74	35,55	2
**TT112**	**2019**	4,34	1,91	3,27	16,52	0,34	26,38	38,92	2
**TT113**	**2019**	4,82	2,00	3,67	18,89	0,27	29,65	43,44	4
**TT115**	**2019**	4,96	2,10	3,77	19,24	0,31	30,38	35,30	2
**TT116**	**2019**	5,41	1,48	3,66	13,58	0,29	24,42	37,71	4
**TT117**	**2019**	4,34	1,92	2,67	16,40	0,18	25,50	36,07	2
**TT118**	**2019**	5,54	2,06	3,46	18,69	0,25	29,99	39,20	2
**TT119**	**2019**	5,13	1,85	3,44	15,30	0,29	26,00	35,42	2
**TT120**	**2019**	4,99	1,44	3,26	15,55	0,24	25,48	42,58	2
**TT121**	**2019**	4,30	1,86	2,84	17,54	0,20	26,74	41,97	5
**TT122**	**2019**	5,54	1,87	3,61	19,35	0,32	30,69	29,71	1
**TT123**	**2019**	4,70	1,82	3,40	16,37	0,22	26,51	44,58	5
**TT124**	**2019**	4,22	1,39	3,15	15,25	0,22	24,23	40,47	5
**TT125**	**2019**	4,72	2,00	3,37	18,90	0,26	29,24	33,34	2
**TT126**	**2019**	5,63	1,35	3,86	15,24	0,37	26,45	34,12	2
**TT127**	**2019**	5,37	1,50	3,30	14,17	0,22	24,56	50,17	5
**TT128**	**2019**	5,02	2,08	3,37	19,68	0,24	30,39	38,39	2
**TT129**	**2019**	4,76	1,49	2,98	14,20	0,20	23,62	50,42	5
**TT130**	**2019**	5,05	2,08	3,22	17,21	0,27	27,84	34,40	2

**TT131**	**2019**	4,28	1,77	1,84	14,88	0,19	22,95	33,99	2
**TT132**	**2019**	4,51	2,21	2,97	18,21	0,19	28,09	37,18	2
**TT133**	**2019**	4,34	1,80	2,67	15,35	0,18	24,34	42,37	5
**TT134**	**2019**	5,09	1,86	3,58	17,64	0,23	28,40	37,13	2
**TT135**	**2019**	5,44	2,56	3,37	17,89	0,22	29,48	29,52	2
**TT136**	**2019**	5,40	2,01	3,60	17,88	0,18	29,07	36,92	4
**TT137**	**2019**	5,17	2,29	3,43	18,00	0,15	29,04	30,22	2
**TT138**	**2019**	4,51	1,94	3,37	18,56	0,23	28,61	29,86	2
**TT139**	**2019**	4,49	1,64	3,13	15,98	0,15	25,39	43,77	5
**TT140**	**2019**	5,49	1,55	3,90	16,72	0,21	27,87	43,86	4
**TT141**	**2019**	4,84	1,48	3,49	15,45	0,20	25,47	37,49	2
**TT142**	**2019**	4,20	1,64	2,91	13,96	0,19	22,89	47,80	5
**TT143**	**2019**	3,89	1,38	2,57	15,11	0,14	23,09	45,28	5
**TT144**	**2019**	5,58	1,66	3,53	16,68	0,16	27,60	49,07	5
**TT145**	**2019**	4,80	1,46	2,91	14,51	0,13	23,80	47,55	5
**TT146**	**2019**	3,62	1,19	2,51	11,54	0,14	19,00	51,16	5
**TT147**	**2019**	5,13	2,26	3,79	18,50	0,21	29,89	34,08	2
**TT148**	**2019**	4,19	1,07	4,10	12,56	0,19	22,12	46,78	5
**TT149**	**2019**	5,46	1,55	3,29	13,53	0,20	24,03	45,25	5
**TT150**	**2019**	4,46	1,57	2,86	11,62	0,10	20,62	52,04	5
**TT151**	**2019**	4,65	1,93	2,82	18,85	0,11	28,36	35,48	2
**TT152**	**2019**	5,55	1,53	4,06	17,62	0,13	28,88	42,70	4
**TT153**	**2019**	4,64	2,10	2,95	17,85	0,11	27,65	40,58	4
**TT154**	**2019**	5,12	1,81	3,72	17,72	0,12	28,48	37,50	4
**TT155**	**2019**	4,87	1,82	3,15	16,31	0,13	26,28	35,49	2
**TT156**	**2019**	4,52	2,01	2,75	16,02	0,13	25,43	39,38	4
**TT157**	**2019**	5,10	2,26	3,03	19,00	0,11	29,51	39,19	4
**TT158**	**2019**	4,36	1,48	2,71	13,20	0,12	21,87	42,36	5
**TT159**	**2019**	5,96	2,28	3,89	20,28	0,11	32,52	42,18	4
**TT160**	**2019**	5,15	1,95	3,25	16,92	0,13	27,40	40,96	2
**TT161**	**2019**	5,75	1,96	3,90	17,55	0,15	29,30	42,78	4
**TT162**	**2019**	5,53	1,94	3,81	19,06	0,15	30,49	36,78	2
**TT163**	**2019**	4,40	2,01	2,79	14,35	0,12	23,67	36,01	2
**TT164**	**2019**	4,37	1,76	2,84	16,99	0,11	26,07	42,20	5
**TT165**	**2019**	4,79	1,83	3,67	15,56	0,11	25,96	44,96	4
**TT166**	**2019**	4,43	2,23	2,54	17,02	0,10	26,32	27,24	2
**TT167**	**2019**	5,99	2,50	3,97	20,74	0,14	33,35	30,33	1
**TT168**	**2019**	5,04	1,68	3,59	17,09	0,09	27,49	39,80	2
**TT169**	**2019**	5,53	1,64	3,35	17,85	0,10	28,46	39,27	4
**TT170**	**2019**	4,17	1,26	2,84	11,55	0,07	19,89	48,50	5
**TT171**	**2019**	4,83	2,18	3,28	21,89	0,10	32,28	33,19	2
**TT172**	**2019**	4,24	1,82	3,33	13,04	0,09	22,52	28,57	2
**TT173**	**2019**	4,81	1,90	2,75	16,52	0,10	26,08	40,13	5
**TT174**	**2019**	5,52	2,38	3,28	19,62	0,13	30,94	31,69	1
**TT175**	**2019**	5,39	1,50	3,48	12,35	0,11	22,83	50,11	5
**TT176**	**2019**	5,09	1,51	3,52	14,81	0,10	25,04	48,22	5

**TT177**	**2019**	4,37	1,48	3,28	13,72	0,12	22,96	41,76	5
**TT178**	**2019**	4,69	1,67	3,93	18,61	0,10	29,00	39,39	4
**TT179**	**2019**	5,37	2,26	3,37	16,79	0,14	27,92	30,19	2
**TT180**	**2019**	5,18	1,75	3,25	16,16	0,14	26,49	43,92	5
**TT181**	**2019**	4,89	1,76	3,48	15,03	0,14	25,30	47,91	5
**TT182**	**2019**	4,88	2,49	3,41	20,25	0,14	31,17	29,27	1
**TT183**	**2019**	4,74	1,77	3,21	16,46	0,12	26,30	42,06	4
**TT184**	**2019**	4,89	1,78	3,10	14,20	0,11	24,07	45,89	5
**TT185**	**2019**	5,42	1,50	4,12	14,99	0,17	26,20	42,73	4
**TT186**	**2019**	4,79	1,62	3,48	17,42	0,11	27,41	50,37	4
**TT187**	**2019**	4,24	1,63	3,26	18,59	0,12	27,84	37,02	2
**TT188**	**2019**	5,34	1,63	3,43	15,90	0,09	26,39	46,61	4
**TT189**	**2019**	4,09	2,03	3,10	17,02	0,11	26,35	40,12	4
**TT190**	**2019**	4,88	2,31	3,16	18,98	0,11	29,44	35,02	2
**TT191**	**2019**	3,74	1,91	2,65	17,41	0,12	25,81	38,22	2
**TT192**	**2019**	4,73	1,65	3,40	15,58	0,10	25,46	40,12	2
**TT193**	**2019**	4,88	1,64	3,29	15,25	0,11	25,16	41,57	5
**TT194**	**2019**	4,18	1,48	3,04	12,77	0,09	21,58	44,94	5
**TT195**	**2019**	5,48	2,14	3,96	19,97	0,12	31,67	37,31	2
**TT196**	**2019**	4,48	1,66	3,62	16,74	0,14	26,64	41,55	2
**TT197**	**2019**	4,82	2,28	3,13	20,92	0,10	31,26	38,18	2
**TT198**	**2019**	5,94	2,20	4,10	21,03	0,15	33,42	36,41	2
**TT199**	**2019**	5,00	1,38	3,72	17,40	0,12	27,61	40,87	4
**TT200**	**2019**	4,85	1,49	3,35	13,36	0,09	23,15	41,20	5
**TT201**	**2019**	4,27	1,39	2,69	14,80	0,12	23,28	44,52	5
**TT202**	**2019**	4,34	1,93	3,51	16,39	0,10	26,27	33,87	2
**TT203**	**2019**	4,35	1,50	3,50	14,45	0,10	23,90	38,89	2
**TT204**	**2019**	4,44	1,80	3,25	15,96	0,10	25,54	40,25	2
**TT205**	**2019**	4,51	2,30	3,12	16,38	0,13	26,44	35,07	2
**TT206**	**2019**	5,58	3,34	3,44	25,52	0,13	38,01	32,90	1
**TT207**	**2019**	4,46	1,59	2,99	12,94	0,08	22,07	47,70	5
**TT208**	**2019**	4,35	2,37	2,80	22,74	0,10	32,36	38,99	3
**TT209**	**2019**	4,52	1,86	3,74	14,51	0,11	24,74	33,34	2
**TT210**	**2019**	4,64	1,74	3,60	16,52	0,11	26,62	45,27	5
**TT211**	**2019**	4,10	1,02	2,73	10,71	0,08	18,65	47,94	5
**TT212**	**2019**	4,00	2,02	2,40	14,78	0,08	23,27	33,31	2
**TT213**	**2019**	6,65	2,92	4,59	20,92	0,17	35,24	26,90	1
**TT214**	**2019**	4,94	1,77	3,94	18,67	0,11	29,43	41,25	4
**TT215**	**2019**	4,89	2,15	3,24	15,63	0,10	26,02	37,83	2
**TT216**	**2019**	4,47	2,18	3,21	19,01	0,19	29,06	35,22	2
**TT217**	**2019**	3,96	1,44	3,02	15,33	0,13	23,87	42,58	5
**TT218**	**2019**	4,86	1,75	3,28	16,48	0,11	26,49	45,17	4
**TT219**	**2019**	5,79	1,94	3,64	20,07	0,10	31,54	39,77	3
**TT220**	**2019**	5,79	2,19	4,23	16,93	0,13	29,28	30,26	2
**TT221**	**2019**	4,81	2,13	3,30	16,41	0,11	26,77	39,97	4
**TT222**	**2019**	4,71	2,70	3,03	18,01	0,10	28,55	35,35	2

**TT223**	**2019**	3,83	1,01	2,39	10,51	0,07	17,82	49,52	5
**TT224**	**2019**	4,20	2,04	3,21	18,68	0,10	28,24	36,71	2
**TT225**	**2019**	4,32	1,63	3,12	13,22	0,08	22,37	39,74	2
**TT226**	**2019**	3,30	1,50	2,05	12,98	0,08	19,90	34,29	2
**TT227**	**2019**	3,85	2,32	3,08	22,75	0,11	32,11	35,44	1
**TT228**	**2019**	3,67	1,40	2,66	10,73	0,10	18,57	48,25	5
**TT1**	**2014**	8,04	4,79	3,00	16,45	0,05	32,34	36,02	1
**TT2**	**2014**	6,74	3,40	2,59	14,96	0,06	27,75	37,27	1
**TT3**	**2014**	8,11	4,06	3,53	19,12	0,05	34,87	43,72	1
**TT4**	**2014**	10,05	3,31	3,16	13,76	0,06	30,34	47,29	3
**TT5**	**2014**	6,29	3,46	2,72	13,57	0,05	26,09	47,66	3
**TT6**	**2014**	5,35	3,63	1,50	16,62	0,03	27,13	43,19	1
**TT7**	**2014**	8,32	4,09	2,72	15,83	0,04	31,00	49,92	3
**TT8**	**2014**	8,52	4,03	2,68	18,00	0,06	33,28	47,75	3
**TT9**	**2014**	5,89	3,91	2,35	16,37	0,07	28,58	40,65	2
**TT10**	**2014**	7,96	3,00	2,52	13,39	0,08	26,96	47,74	4
**TT11**	**2014**	8,25	2,98	3,33	13,89	0,09	28,53	46,51	4
**TT12**	**2014**	8,34	4,07	3,04	17,03	0,10	32,58	44,67	1
**TT13**	**2014**	6,70	3,33	2,19	13,85	0,08	26,15	51,69	4
**TT14**	**2014**	6,41	2,92	2,24	8,55	0,05	20,17	57,23	5
**TT15**	**2014**	8,44	3,47	3,35	16,24	0,07	31,57	40,09	1
**TT16**	**2014**	6,62	3,55	2,32	15,87	0,06	28,43	52,31	3
**TT17**	**2014**	6,86	2,80	2,60	13,24	0,06	25,56	50,57	4
**TT18**	**2014**	5,12	3,04	2,61	13,07	0,04	23,88	45,37	2
**TT19**	**2014**	7,27	2,89	2,37	13,42	0,06	26,01	52,16	4
**TT20**	**2014**	7,50	3,45	3,27	15,52	0,08	29,82	49,73	3
**TT21**	**2014**	7,49	4,16	2,92	18,30	0,06	32,93	34,33	1
**TT22**	**2014**	7,96	3,88	2,33	15,04	0,08	29,29	48,69	3
**TT23**	**2014**	5,38	2,71	2,01	13,97	0,06	24,12	50,16	4
**TT24**	**2014**	7,13	3,58	2,20	15,37	0,08	28,36	47,43	3
**TT25**	**2014**	8,40	2,88	2,90	12,50	0,07	26,74	43,93	4
**TT26**	**2014**	7,28	4,12	2,98	17,68	0,07	32,13	45,16	3
**TT27**	**2014**	6,75	3,97	2,97	16,12	0,00	29,82	42,79	3
**TT28**	**2014**	7,63	3,11	2,75	15,29	0,04	28,81	45,63	3
**TT29**	**2014**	6,26	3,97	2,51	17,96	0,12	30,82	35,04	1
**TT30**	**2014**	8,03	3,58	2,41	12,63	0,08	26,72	57,71	4
**TT31**	**2014**	6,22	3,21	2,01	10,94	0,00	22,38	54,41	5
**TT32**	**2014**	8,10	3,96	2,99	17,11	0,08	32,26	41,51	1
**TT33**	**2014**	6,05	3,32	2,96	15,04	0,14	27,51	39,08	1
**TT34**	**2014**	6,82	2,63	1,87	8,92	0,07	20,30	54,14	5
**TT35**	**2014**	3,91	2,30	1,72	14,39	0,12	22,44	48,06	3
**TT36**	**2014**	5,36	2,89	2,34	11,69	0,06	22,33	47,10	4
**TT37**	**2014**	3,68	1,75	1,62	8,86	0,16	16,07	50,08	5
**TT38**	**2014**	5,82	3,07	1,90	13,46	0,07	24,32	49,28	4
**TT39**	**2014**	7,72	4,26	3,21	18,45	0,09	33,74	41,77	1
**TT40**	**2014**	6,13	3,80	2,69	13,20	0,10	25,92	38,32	1

**TT41**	**2014**	6,26	3,21	2,72	14,27	0,08	26,55	49,49	3
**TT42**	**2014**	3,95	2,81	1,48	11,02	0,07	19,33	44,88	2
**TT43**	**2014**	7,80	2,65	2,89	11,18	0,10	24,61	54,39	4
**TT44**	**2014**	6,66	2,92	2,29	12,55	0,07	24,49	52,39	4
**TT45**	**2014**	6,73	3,39	2,32	15,00	0,07	27,50	44,01	1
**TT46**	**2014**	7,57	3,25	2,16	12,63	0,07	25,68	51,17	4
**TT47**	**2014**	4,94	2,62	1,92	10,84	0,06	20,39	55,13	4
**TT48**	**2014**	6,20	3,53	2,25	18,34	0,05	30,38	42,75	1
**TT49**	**2014**	6,32	4,12	2,51	15,95	0,05	28,95	35,22	1
**TT50**	**2014**	5,43	2,66	2,47	13,77	0,06	24,40	45,40	3
**TT51**	**2014**	5,31	3,33	2,03	17,26	0,07	28,00	40,66	1
**TT52**	**2014**	7,56	2,86	2,57	9,36	0,09	22,44	51,27	4
**TT53**	**2014**	6,31	2,85	2,27	14,20	0,06	25,69	42,18	2
**TT54**	**2014**	9,34	2,92	3,18	16,48	0,06	31,98	51,13	3
**TT55**	**2014**	6,46	2,63	2,47	14,03	0,05	25,65	47,70	3
**TT56**	**2014**	7,14	2,76	2,55	13,29	0,06	25,81	50,18	4
**TT57**	**2014**	6,05	3,07	2,47	15,02	0,06	26,67	50,49	4
**TT58**	**2014**	5,65	2,76	2,42	15,37	0,10	26,30	42,92	3
**TT59**	**2014**	6,09	2,84	2,34	9,70	0,08	21,04	52,04	5
**TT60**	**2014**	8,00	3,76	2,79	14,57	0,06	29,17	56,48	4
**TT61**	**2014**	8,06	3,39	2,72	14,36	0,07	28,60	42,42	3
**TT62**	**2014**	7,36	2,86	2,74	14,14	0,06	27,16	52,81	4
**TT63**	**2014**	5,43	3,18	2,04	12,70	0,05	23,41	42,37	2
**TT64**	**2014**	7,55	2,78	2,83	13,21	0,06	26,42	49,86	4
**TT65**	**2014**	6,95	2,91	3,23	12,76	0,07	25,93	44,74	4
**TT66**	**2014**	6,11	2,54	2,30	10,59	0,08	21,61	42,69	2
**TT67**	**2014**	5,90	3,07	2,09	15,49	0,05	26,60	47,19	4
**TT68**	**2014**	4,70	2,38	2,19	13,24	0,12	22,64	46,65	4
**TT69**	**2014**	5,96	2,41	2,16	13,76	0,05	24,35	41,29	2
**TT70**	**2014**	7,14	3,09	2,42	9,99	0,07	22,70	48,53	4
**TT71**	**2014**	5,47	2,80	1,96	13,72	0,06	23,99	51,21	4
**TT72**	**2014**	5,37	1,84	2,30	9,10	0,09	18,71	53,76	5
**TT73**	**2014**	7,48	3,08	2,59	16,11	0,06	29,32	51,27	3
**TT74**	**2014**	5,70	2,82	2,15	11,51	0,06	22,24	53,92	4
**TT75**	**2014**	7,88	2,79	2,91	14,18	0,07	27,83	47,32	3
**TT76**	**2014**	6,37	2,60	2,19	12,86	0,06	24,08	39,75	1
**TT77**	**2014**	4,95	2,31	2,42	13,74	0,12	23,55	44,24	3
**TT78**	**2014**	5,48	3,11	2,22	15,33	0,07	26,21	39,64	1
**TT79**	**2014**	6,46	3,59	2,91	12,01	0,09	25,07	38,59	3
**TT80**	**2014**	5,93	2,55	2,15	13,16	0,10	23,90	44,39	3
**TT81**	**2014**	6,53	2,32	2,27	13,55	0,06	24,73	48,10	3
**TT82**	**2014**	7,06	3,59	2,81	13,79	0,06	27,31	49,00	3
**TT83**	**2014**	6,31	2,52	2,52	11,13	0,10	22,58	47,43	4
**TT84**	**2014**	8,11	2,83	2,86	11,48	0,09	25,36	44,57	3
**TT85**	**2014**	8,77	3,19	2,83	15,55	0,07	30,41	43,32	3
**TT86**	**2014**	8,83	3,04	3,02	14,63	0,09	29,61	40,08	2

**TT87**	**2014**	7,17	3,44	2,68	16,05	0,09	29,43	35,45	1
**TT88**	**2014**	5,50	2,75	2,40	12,98	0,07	23,70	35,42	1
**TT89**	**2014**	6,44	3,09	2,42	14,14	0,11	26,20	49,79	4
**TT90**	**2014**	5,04	2,40	2,24	12,74	0,09	22,52	51,48	4
**TT91**	**2014**	5,70	3,31	2,35	15,11	0,13	26,60	37,37	1
**TT92**	**2014**	6,35	2,85	2,15	12,72	0,07	24,15	41,94	4
**TT93**	**2014**	8,16	3,33	2,91	15,95	0,10	30,46	43,80	3
**TT94**	**2014**	7,89	2,65	2,74	12,42	0,11	25,81	50,50	4
**TT95**	**2014**	4,20	1,99	1,63	10,28	0,05	18,15	45,05	4
**TT96**	**2014**	6,38	2,54	2,55	10,73	0,10	22,30	56,57	5
**TT97**	**2014**	8,97	3,03	3,34	14,78	0,11	30,23	47,25	3
**TT98**	**2014**	4,79	2,15	2,12	9,53	0,10	18,69	53,75	5
**TT99**	**2014**	5,91	2,55	2,24	12,82	0,10	23,62	47,08	4
**TT100**	**2014**	7,48	3,44	3,24	16,46	0,14	30,77	33,53	2
**TT101**	**2014**	7,76	3,67	3,17	14,18	0,11	28,89	39,24	2
**TT102**	**2014**	5,07	2,46	2,06	11,66	0,10	21,35	45,24	5
**TT103**	**2014**	6,70	3,04	2,53	12,83	0,12	25,22	45,60	2
**TT104**	**2014**	5,63	2,60	2,80	12,75	0,11	23,88	36,64	2
**TT105**	**2014**	6,38	3,23	2,59	15,00	0,16	27,36	40,59	2
**TT106**	**2014**	4,10	2,29	1,84	13,32	0,10	21,65	37,97	2
**TT107**	**2014**	2,99	3,29	2,19	13,05	0,13	21,65	52,62	4
**TT108**	**2014**	4,13	3,93	3,31	14,56	0,19	26,13	45,39	4
**TT109**	**2014**	3,67	2,82	2,32	12,08	0,08	20,96	50,27	5
**TT110**	**2014**	3,47	3,26	2,38	14,90	0,10	24,11	46,47	4
**TT111**	**2014**	5,43	2,30	2,58	14,91	0,09	25,32	44,56	2
**TT112**	**2014**	2,88	2,41	2,68	14,37	0,09	22,42	45,82	2
**TT113**	**2014**	3,26	2,65	2,58	12,30	0,10	20,89	48,97	4
**TT115**	**2014**	2,89	2,66	2,25	12,34	0,09	20,24	44,87	2
**TT116**	**2014**	3,55	3,17	3,22	13,65	0,10	23,70	44,46	4
**TT117**	**2014**	3,08	3,10	2,92	14,09	0,40	23,59	44,25	2
**TT118**	**2014**	3,57	3,08	2,32	13,02	0,41	22,40	47,12	2
**TT119**	**2014**	4,04	2,10	2,24	9,77	0,09	18,24	38,99	2
**TT120**	**2014**	3,12	2,55	2,69	13,59	0,10	22,05	47,86	2
**TT121**	**2014**	2,88	2,88	2,06	12,35	0,07	20,23	49,96	5
**TT122**	**2014**	3,24	3,53	2,86	16,17	0,13	25,94	36,34	1
**TT123**	**2014**	2,78	2,48	2,34	11,02	0,07	18,69	50,16	5
**TT124**	**2014**	3,04	2,59	2,35	12,15	0,07	20,20	52,12	5
**TT125**	**2014**	2,83	3,40	2,40	14,37	0,08	23,08	40,27	2
**TT126**	**2014**	2,33	1,50	2,07	8,72	0,10	14,71	39,14	2
**TT127**	**2014**	3,17	2,59	2,09	10,11	0,07	18,01	57,72	5
**TT128**	**2014**	5,45	2,84	2,14	13,99	0,09	24,52	44,60	2
**TT129**	**2014**	2,88	2,50	1,79	10,82	0,06	18,05	58,52	5
**TT130**	**2014**	2,50	2,46	2,02	11,05	0,09	18,11	42,08	2
**TT131**	**2014**	1,79	2,23	0,80	9,72	0,08	14,62	37,65	2
**TT132**	**2014**	3,03	3,35	2,74	14,62	0,10	23,83	43,88	2
**TT133**	**2014**	2,86	2,52	1,90	10,37	0,09	17,74	50,25	5

**TT134**	**2014**	1,68	2,20	1,95	9,30	0,09	15,23	45,39	2
**TT135**	**2014**	1,71	2,94	2,20	13,78	0,10	20,73	51,92	2
**TT136**	**2014**	2,06	3,07	2,34	12,97	0,12	20,57	49,96	4
**TT137**	**2014**	1,76	2,96	2,25	13,57	0,05	20,58	56,96	2
**TT138**	**2014**	1,38	2,45	2,08	12,98	0,05	18,94	50,43	2
**TT139**	**2014**	1,53	1,85	1,98	9,52	0,05	14,94	50,49	5
**TT140**	**2014**	2,10	2,61	2,88	12,78	0,08	20,44	46,26	4
**TT141**	**2014**	1,70	2,19	2,52	11,13	0,04	17,58	43,64	2
**TT142**	**2014**	1,68	2,35	1,89	9,25	0,05	15,22	55,69	5
**TT143**	**2014**	1,62	2,14	1,86	13,03	0,06	18,70	52,13	5
**TT144**	**2014**	2,30	2,87	2,52	12,96	0,04	20,68	56,21	5
**TT145**	**2014**	2,00	2,00	1,87	9,60	0,03	15,50	50,50	5
**TT146**	**2014**	1,72	2,46	1,74	10,51	0,03	16,45	57,55	5
**TT147**	**2014**	6,83	3,66	3,10	15,15	0,05	28,80	41,01	2
**TT148**	**2014**	7,08	2,66	2,93	12,90	0,07	25,64	53,78	5
**TT149**	**2014**	7,02	3,07	2,39	13,17	0,66	26,31	51,20	5
**TT150**	**2014**	5,37	2,66	1,88	14,33	0,62	24,86	39,39	5
**TT151**	**2014**	6,18	2,58	2,63	10,98	0,50	22,86	47,35	2
**TT152**	**2014**	6,03	1,82	2,47	10,61	0,52	21,44	52,49	4
**TT153**	**2014**	4,31	2,78	1,59	14,23	0,43	23,35	52,10	4
**TT154**	**2014**	5,48	2,56	2,53	11,80	0,44	22,81	49,04	4
**TT155**	**2014**	5,63	2,95	2,23	13,89	0,76	25,45	40,88	2
**TT156**	**2014**	7,78	3,84	2,40	15,00	1,14	30,17	46,84	4
**TT157**	**2014**	5,58	2,55	2,02	11,59	0,81	22,55	50,18	4
**TT158**	**2014**	3,63	1,73	1,42	8,94	0,75	16,46	52,18	5
**TT159**	**2014**	6,72	2,88	2,37	13,40	0,75	26,12	50,98	4
**TT160**	**2014**	5,09	2,66	1,78	12,03	0,73	22,28	46,76	2
**TT161**	**2014**	5,61	2,15	2,31	10,38	0,64	21,10	56,07	4
**TT162**	**2014**	5,14	2,34	1,94	12,05	0,67	22,14	43,08	2
**TT163**	**2014**	4,40	2,58	1,88	9,84	1,28	19,98	40,95	2
**TT164**	**2014**	4,85	2,44	1,77	11,64	0,78	21,47	51,73	5
**TT165**	**2014**	5,71	2,74	2,31	11,70	1,05	23,50	53,92	4
**TT166**	**2014**	4,73	3,09	1,43	13,89	0,89	24,03	40,13	2
**TT167**	**2014**	5,80	3,17	2,48	14,19	1,03	26,67	38,84	1
**TT168**	**2014**	4,97	2,64	2,19	12,95	0,96	23,71	45,64	2
**TT169**	**2014**	6,11	2,62	2,20	12,79	0,93	24,65	47,06	4
**TT170**	**2014**	4,97	2,01	1,77	8,59	0,75	18,09	57,36	5
**TT171**	**2014**	5,54	2,78	2,12	16,49	0,77	27,69	44,02	2
**TT172**	**2014**	5,78	2,61	2,87	11,99	0,74	24,00	25,97	2
**TT173**	**2014**	6,23	2,68	1,74	10,88	0,92	22,45	50,20	5
**TT174**	**2014**	6,39	3,60	2,21	15,14	0,87	28,20	37,88	1
**TT175**	**2014**	7,36	2,58	2,41	8,86	0,86	22,07	56,18	5
**TT176**	**2014**	7,55	2,83	2,47	11,64	0,92	25,42	53,32	5
**TT177**	**2014**	6,09	2,35	2,35	11,37	0,88	23,04	52,34	5
**TT178**	**2014**	6,63	2,62	2,68	14,28	0,85	27,07	47,93	4
**TT179**	**2014**	7,17	3,56	2,33	13,60	0,90	27,56	42,95	2

**TT180**	**2014**	7,28	3,04	2,25	12,45	0,89	25,90	52,18	5
**TT181**	**2014**	6,75	2,94	2,40	11,16	0,91	24,16	56,05	5
**TT182**	**2014**	5,88	3,35	2,54	15,32	0,79	27,88	37,77	1
**TT183**	**2014**	7,12	3,13	2,37	11,87	1,17	25,67	50,85	4
**TT184**	**2014**	6,75	2,90	2,07	11,02	1,01	23,76	49,48	5
**TT185**	**2014**	8,83	3,14	3,76	13,60	1,26	30,59	45,53	4
**TT186**	**2014**	7,46	3,09	2,75	15,23	1,02	29,55	56,43	4
**TT187**	**2014**	7,43	3,72	2,76	15,49	1,29	30,69	41,89	2
**TT188**	**2014**	6,48	2,66	2,74	13,78	1,17	26,81	48,99	4
**TT189**	**2014**	5,73	2,92	2,25	12,54	1,26	24,70	47,02	4
**TT190**	**2014**	4,62	2,60	2,25	13,80	1,11	24,39	42,97	2
**TT191**	**2014**	3,99	2,66	1,80	11,85	1,14	21,44	42,98	2
**TT192**	**2014**	6,12	2,76	2,20	12,05	1,01	24,14	46,53	2
**TT193**	**2014**	6,00	2,43	2,02	10,77	1,02	22,25	50,37	5
**TT194**	**2014**	4,86	2,18	1,87	10,11	0,92	19,94	52,78	5
**TT195**	**2014**	4,85	2,10	2,31	12,07	0,93	22,27	44,76	2
**TT196**	**2014**	5,72	2,85	2,57	13,15	1,09	25,37	44,77	2
**TT197**	**2014**	4,59	3,26	1,63	13,81	0,98	24,27	46,28	2
**TT198**	**2014**	4,92	2,31	1,92	11,41	0,92	21,48	43,21	2
**TT199**	**2014**	4,72	1,90	2,15	12,31	0,96	22,04	51,59	4
**TT200**	**2014**	6,15	2,17	2,53	10,06	1,06	21,96	49,08	5
**TT201**	**2014**	5,27	2,12	2,09	11,43	1,03	21,93	50,94	5
**TT202**	**2014**	6,25	3,29	2,88	13,62	1,07	27,11	37,96	2
**TT203**	**2014**	5,31	2,56	2,61	11,06	0,99	22,53	42,93	2
**TT204**	**2014**	5,00	2,69	2,16	11,79	0,89	22,52	43,42	2
**TT205**	**2014**	4,02	2,46	1,75	11,07	0,88	20,18	40,05	2
**TT206**	**2014**	5,09	3,83	2,14	17,83	1,39	30,28	40,37	1
**TT207**	**2014**	5,49	2,43	1,96	9,92	1,27	21,08	55,31	5
**TT208**	**2014**	4,94	3,18	2,03	17,73	1,35	29,24	45,11	3
**TT209**	**2014**	4,78	2,50	2,70	13,69	0,08	23,74	42,89	2
**TT210**	**2014**	3,10	1,60	2,29	12,17	0,07	19,24	52,17	5
**TT211**	**2014**	6,23	2,28	2,43	11,98	0,07	22,98	52,08	5
**TT212**	**2014**	1,76	1,15	1,38	9,32	0,08	13,69	38,25	2
**TT213**	**2014**	7,20	3,71	3,30	14,76	0,11	29,09	32,71	1
**TT214**	**2014**	5,91	2,89	3,31	16,46	0,09	28,66	47,67	4
**TT215**	**2014**	7,17	3,36	2,51	12,96	0,09	26,09	43,47	2
**TT216**	**2014**	4,74	2,67	2,35	12,88	0,07	22,71	39,78	2
**TT217**	**2014**	4,58	2,41	2,31	13,21	0,08	22,59	52,05	5
**TT218**	**2014**	6,03	3,17	2,57	13,34	0,07	25,19	54,30	4
**TT219**	**2014**	8,21	2,71	2,82	14,71	0,08	28,52	44,33	3
**TT220**	**2014**	5,53	3,05	2,98	13,43	0,10	25,09	35,07	2
**TT221**	**2014**	5,84	2,88	2,10	14,26	0,11	25,19	49,65	4
**TT222**	**2014**	5,87	3,20	2,24	13,42	0,11	24,84	42,40	2
**TT223**	**2014**	6,01	2,18	2,33	10,99	0,08	21,60	52,55	5
**TT224**	**2014**	6,03	3,45	2,69	16,48	0,12	28,77	43,55	2
**TT225**	**2014**	6,97	3,03	2,99	12,56	0,11	25,66	42,62	2

**TT226**	**2014**	4,34	2,89	1,52	8,71	0,14	17,60	36,10	2
**TT227**	**2014**	4,65	3,52	1,95	17,07	0,10	27,28	34,93	1
**TT228**	**2014**	7,50	3,72	3,04	14,37	0,13	28,76	48,54	5
**min 2014**		**1,38**	**1,15**	**0,80**	**8,55**	**0,00**	**13,69**	**25,97**	
**min 2019**		**3,30**	**1,01**	**1,84**	**10,51**	**0,06**	**17,82**	**26,90**	
**min total**		**1,38**	**1,01**	**0,80**	**8,55**	**0,00**	**13,69**	**25,97**	
**max 2014**		**10,05**	**4,79**	**3,76**	**19,12**	**1,39**	**34,87**	**58,52**	
**max 2019**		**8,39**	**3,88**	**5,64**	**28,94**	**0,43**	**45,18**	**53,47**	
**max total**		**10,05**	**4,79**	**5,64**	**28,94**	**1,39**	**45,18**	**58,52**	
**mean 2014**		**5,67**	**2,91**	**2,40**	**13,19**	**0,31**	**24,48**	**46,47**	
**mean 2019**		**5,48**	**2,16**	**3,75**	**19,07**	**0,15**	**30,61**	**40,44**	
**mean total**		**5,58**	**2,53**	**3,07**	**16,12**	**0,23**	**27,53**	**43,47**	
**sd 2014**		**1,82**	**0,57**	**0,45**	**2,25**	**0,39**	**4,12**	**5,93**	
**sd 2019**		**1,04**	**0,56**	**0,75**	**4,13**	**0,07**	**5,93**	**5,39**	
**sd total**		**1,49**	**0,67**	**0,91**	**4,43**	**0,29**	**5,94**	**6,40**	
