# Supplementary figures and images for: Variation in the Content and Composition of Tocols in a Wheat Population
Source: Foods. 2022 May 5;11(9):1343. doi: 10.3390/foods11091343 (PMC9105132; doi:10.3390/foods11091343)

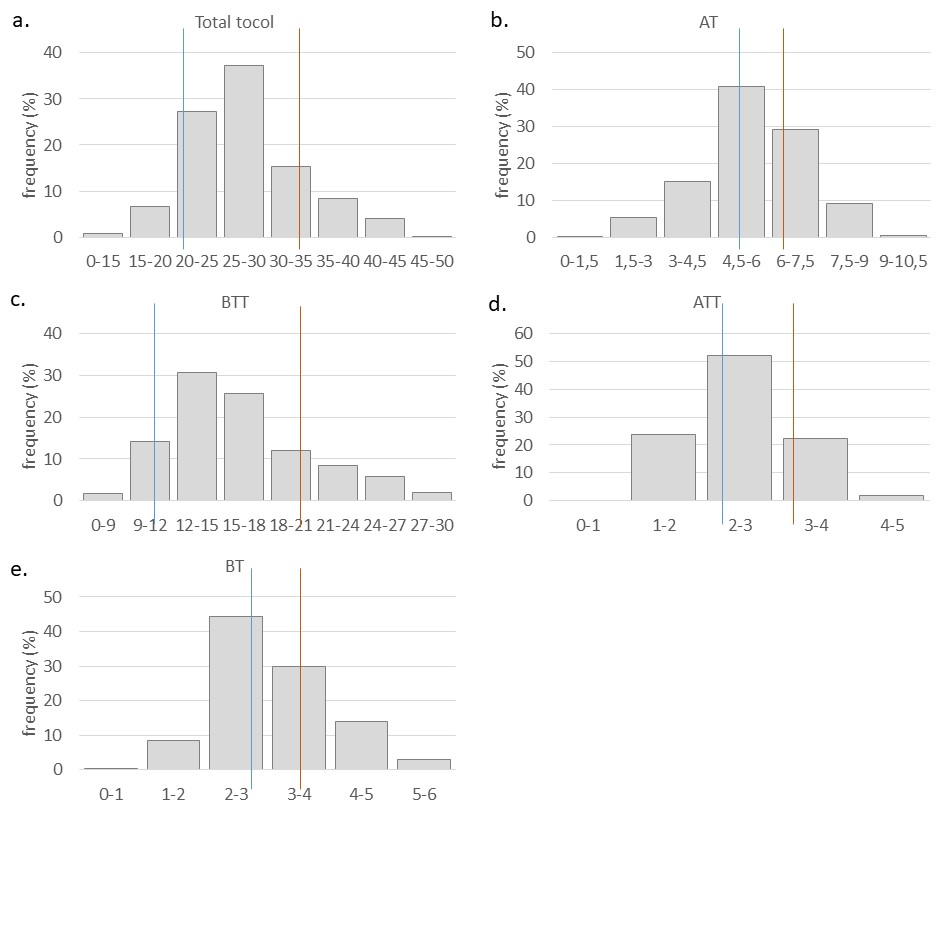

Supplement: Supplementary file 1 [file foods-11-01343-s001.zip › Figure S1.jpg]

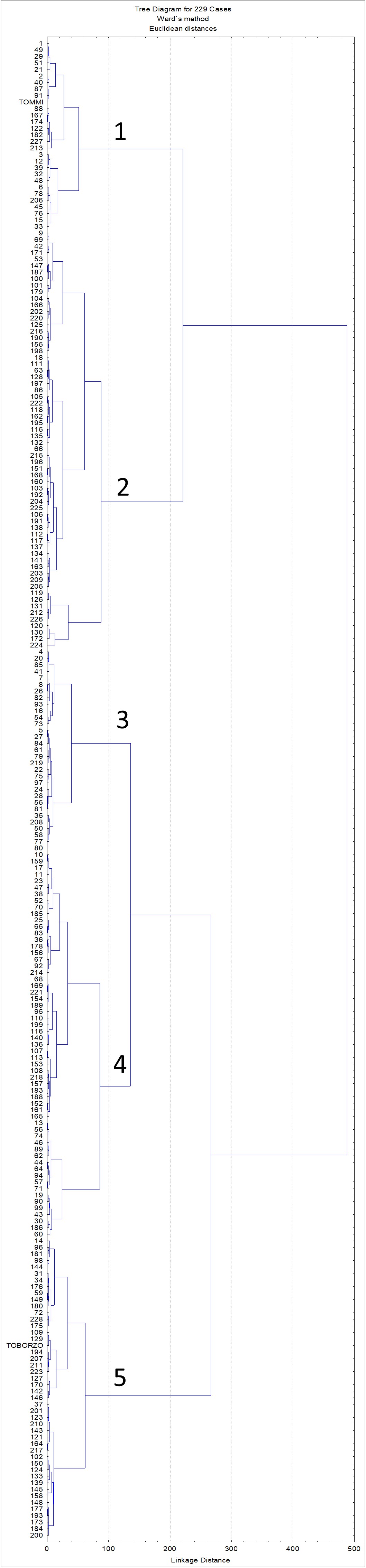

Supplement: Supplementary file 1 [file foods-11-01343-s001.zip › Figure S2.jpg]
